# Strategies for engaging older adults and informal caregivers in health policy development: A scoping review

**DOI:** 10.1186/s12961-024-01107-9

**Published:** 2024-02-19

**Authors:** Opeyemi Rashidat Kolade, Joshua Porat-Dahlerbruch, Rustem Makhmutov, Theo van Achterberg, Moriah Esther Ellen

**Affiliations:** 1 Department of Health Policy and Management, Guilford Glazer Faculty of Business and Management and Faculty of Health Sciences, Ben-Gurion University of the Negev, Be’er Sheva, Israel; 2 Academic Centre for Nursing and Midwifery, Department of Public Health and Primary Care, University of Leuven, KU Leuven, Kapucijnenvoer 35, 3000 Louvain, Belgium; 3 Department of Acute and Tertiary Care, School of Nursing, University of Pittsburgh, 336 Victoria Building, 3500 Victoria Street, Pittsburgh, PA 15261 USA; 4 Institute for Health and Nursing Science, Medical Faculty, Martin Luther University Halle-Wittenberg, Magdeburger Straße 8, 06112 Halle (Saale), Germany; 5 Institute of Health Policy Management and Evaluation, Dalla Lana School of Public Health, University of Toronto, Toronto, Canada

**Keywords:** Older adults, Aged, Informal caregivers, Family carers, Involvement, Participation, Engagement, Policy-making, Decision-making, Health policy development

## Abstract

**Background:**

Care for older adults is high on the global policy agenda. Active involvement of older adults and their informal caregivers in policy-making can lead to cost–effective health and long-term care interventions. Yet, approaches for their involvement in health policy development have yet to be extensively explored. This review maps the literature on strategies for older adults (65+ years) and informal caregivers’ involvement in health policy development.

**Method:**

As part of the European Union TRANS-SENIOR program, a scoping review was conducted using the Joanna Briggs Institute’s methodology. Published and grey literature was searched, and eligible studies were screened. Data were extracted from included studies and analysed using the Multidimensional Framework for Patient and Family Engagement in Health and Healthcare.

**Results:**

A total of 13 engagement strategies were identified from 11 publications meeting the inclusion criteria. They were categorized as “traditional”, “deliberative” and “others”, adopting the World Bank’s categorization of engagement methods. Older adults and informal caregivers are often consulted to elicit opinions and identify priorities. However, their involvement in policy formulation, implementation and evaluation is unclear from the available literature. Findings indicate that older adults and their informal caregivers do not often have equal influence and shared leadership in policy-making.

**Conclusion:**

Although approaches for involving older adults and their informal caregivers’ involvement were synthesized from literature, we found next to no information about their involvement in policy formulation, implementation and evaluation. Findings will guide future research in addressing identified gaps and guide policy-makers in identifying and incorporating engagement strategies to support evidence-informed policy-making processes that can improve health outcomes for older adults/informal caregivers.

**Supplementary Information:**

The online version contains supplementary material available at 10.1186/s12961-024-01107-9.

## Introduction

One in six people worldwide will be over 65 years in 2050 [1]. Ageing is correlated with increased multimorbidity and chronic health needs. This is burdensome to older adults and their informal caregivers and significantly increases health and social care service utilization [2, 3], and the complexity of health and long-term care needs. Informal caregiving and care for older adults have become critical issues of public policy. Informal caregivers of older adults are the mainstay of support for older adults with chronic health conditions [4]. Furthermore, they are potentially at risk for adverse effects on their health and well-being, quality of life and economic security [5]. Therefore, health policy decisions are relevant to older adults and their informal caregivers and impact the healthcare system, highlighting the need to develop responsive health policies [6–8]. One approach to efficacious health policy development is citizen engagement [9].

When citizens are engaged in policy development, policy-makers are better aware of needs and outcomes affecting the target population [8]. Citizen engagement in policy-making can improve instrumental (designed to improve the quality of decision-making), developmental (intended to improve knowledge and capacity of the participants) and democratic (intended to meet transparency, accountability, trust and confidence goals) outcomes [10–12]. As such, involving older adults and their informal caregivers in health policy development improves the legitimacy and transparency of the health policy-making process and can also lead to carefully crafted, relevant policies that improve cost–effective healthcare and long-term care interventions [8, 13, 14]. However, informal caregiver and older adult input in health policy development is limited, and little is known about strategies to engage them in developing policies affecting their lives [15, 16]. Most previous research on engagement at the policy level focuses on the general population, which does not often reflect older adults’ unique and complex social and healthcare needs [17]. Policy-making would benefit from older adult and informal caregiver perspectives, as their involvement has been shown to improve policy sustainability and outcomes [6]. Finally, tangible public health outcomes emerge when active citizen participation in decision-making is promoted [18].

Engaging older adults in health policy development holds substantial public health implications, affecting diverse aspects of healthcare. This impact spans from individual older adults and informal caregivers to the broader societal level, influencing healthcare systems, policies, emerging technologies, healthcare innovation and the overall quality of care. Participation in shaping solutions and emerging health technologies enables the creation of practical and effective solutions alongside those experiencing the issues. For instance, employing collaborative methods such as creative workshops and dilemma games fosters active involvement in co-developing health technologies [19]. Similarly, involvement methods such as patient journey mapping, surveys, workshops, expert panels, user boards, public and patient involvement (PPI) conferences, Delphi methods, living laboratories for technology innovation, stakeholder activities and patient interviews have been employed in the co-development of health services [20].

Most existing research focuses on citizens’ engagement in research. For example, engaging older adults as partners in transitional care research, engaging patients in health research and engaging older adults in healthcare research and planning [21–25]. Previous research has also focused on involvement of older adults/informal caregivers in healthcare decision-making [26, 27] as opposed to older adult/informal caregiver engagement in health policy development. There are a few examples of older adult and informal caregiver engagement in health policy development [17, 28, 29]. However, these works include no overview nor synthesis of methods for engaging older adults and informal caregivers in health policy development. More information on strategies for their engagement is necessary to promote older adult and informal caregiver engagement in health policy development and improve outcomes linked to health policy. This review aims to provide a foundation for older adult and informal caregiver engagement in health policy development by providing an overview of available research evidence on strategies for their engagement in health and well-being policy development. The scope of this study is thus focused specifically on exploring older adult and informal caregiver involvement in government policy development rather than involvement in individual care/healthcare decision-making, and organizational governance/policy-making.

## Methods

### Design

Using the Joanna Briggs Institute (JBI) methodology, we gathered evidence on engagement strategies for older adults and informal caregivers in health policy development, and identified and analysed knowledge gaps [30]. A scoping review protocol was developed to guide the study [31]. Data analysis was guided by the Multidimensional Framework for Patient and Family Engagement in Health and Healthcare by Carman et al. [32] (Additional file 1: Appendix S1), which was influenced by Arnstein’s ladder of participation [33]. The multidimensional framework describes a continuum of engagement (consultation, involvement and partnership/shared leadership) across three levels of the healthcare system (individual care, organizational governance and government policy) and describes factors influencing engagement. This study derives the basis for analysis from the following elements adapted from the framework: the engagement continuum (consultation, involvement, partnership/shared leadership), the level/stage of government policy (agenda setting, policy formulation, policy implementation, policy evaluation) and factors influencing policy-makers to create involvement opportunities, all described in data charting section below. Finally, we were interested in synthesizing data on reported outcomes of engagement.

There are a plethora of potentially relevant frameworks, theories and conceptualizations related to citizen engagement, such as Arnstein’s ladder of citizen participation and the International Association for Public Participation (Iap2) models, among others [33–38]. However, for this study, we opted for the Multidimensional framework by Carman and colleagues. This choice was based on its ability to illustrate different levels of the healthcare system where patients and families can be engaged, a fundamental aspect of our study.

### Search strategy and selection criteria

An initial search of two online databases (PubMed and Embase) was conducted. We analysed the keywords in the title and abstract of retrieved papers and the index terms used to describe the articles. A second search used all identified keywords and index terms across all relevant databases: Health Systems Evidence, Health Evidence, CINAHL, PubMed, and Embase. Thirdly, the reference lists of identified reports and articles were scanned for additional sources. We worked with three librarians for search terms and search strategy refinement. We searched grey literature (Participedia.net and Google) using a combination of indexing (Mesh) terms and the following search key words: (older adult OR aged OR senior) AND (patient participation OR empowerment OR deliberation OR activation) AND (health policy OR Advisory committee OR policy formulation; Additional file 2: Appendix S2). We refined search results from Participedia.net by using filters relating to health and well-being.

Search results were imported into EndNote 20 for de-duplication, then into an online systematic review software, Covidence (www.covidence.org). Titles and abstracts were screened to determine eligibility for full-text review. Results from the grey literature were screened by reading the titles and summaries. All five research team members screened a sample together for eligibility and discussed any doubts and differences. Then all titles and abstracts were screened independently by at least two team members. Disagreements were discussed and resolved through discussion or involving a third team member, and a consensus was reached.

Titles and abstracts of empirical studies, reviews and grey literature reports were included for full-text review if they reported on policy development in the areas of health and well-being, addressed the use or evaluation of a method for engaging older adults and/or informal caregivers in health and well-being policy development, focused on older adults (defined as persons with a minimum age of 65 years; a majority of participants were aged 65 years and above) and/or their informal caregivers, or used proxy words (such as chronically ill, dementia and frail elderly), and addressed policy development at regional, national or international level. In the screening of titles and abstracts, we specifically sought studies that identified participants as either older adults aged 65 years and above, informal caregivers or a combination of both. For full-text screening, we checked for the actual ages and percentage of participants aged 65 years and above. Studies that only included participants younger than 65 years were excluded. Articles with a majority of 65 years and older were included. Corresponding authors of full-text articles were contacted for clarity when the sample population was unclear. Articles were not included in this review when there was no response.

Empirical studies, reviews and grey literature reported in all languages and from the databases’ inception were included. Titles and abstracts of included studies in languages other than English were first screened by a colleague able to read the applicable language to decide on its relevance for extraction. Studies describing older adult and informal caregiver engagement in research or in healthcare decision-making were excluded. We excluded articles describing engagement for organizational governance/policy development. Studies that involved heterogeneous population groups (for example, older adults/informal caregivers and health workers, or government representatives) were excluded. Finally, studies whereby we could not access the full texts were excluded.

### Data charting

Based on the elements of the multidimensional framework for patient and family engagement in health and healthcare [32], a preliminary data charting table (Additional file 3: Appendix S3) was developed and piloted by two team members who also extracted the data. In case of discrepancies, a third team member reviewed for extraction agreement. Data charting followed the following elements – continuum of engagement, stages of government policy development and factors influencing policy-makers to create engagement opportunities. Additionally, data were extracted on outcomes of engagement which we defined as the result of engaging older adults in health policy development.

The engagement continuum is categorized based on how much information flows between parties (policy-maker and citizens), and the level of influence citizens have over decision-making ranging from limited participation, or degrees of tokenism, to a state of collaborative partnership in which citizens share leadership or control decisions. The engagement continuum, according to Carman et al., includes consultation (eliciting opinions about health issues), involvement (when research recommendations influence policy) and partnership/shared leadership (when citizens and policy-makers share equal power and responsibility in decision-making) [32].

Levels of engagement in the framework was modified to “levels/stages of policy development” since we were particularly focused on engagement in policy development. We focused on these stages of the policy cycle: agenda setting (identifying priorities, recognition of issues as a problem demanding public attention), policy formulation (developing and refining policy options for government), policy implementation (activities taken to achieve goals stated in policy statement) and policy evaluation (examination of the effects ongoing policies and public programs have on their targets in terms of the goals they are meant to achieve) [39]. Factors influencing engagement were modified to factors influencing policy-makers to create opportunities for engagement, as this was a gap in the framework this review set out to fill to build on the framework.

## Results

### Study selection

**Fig. 1:**
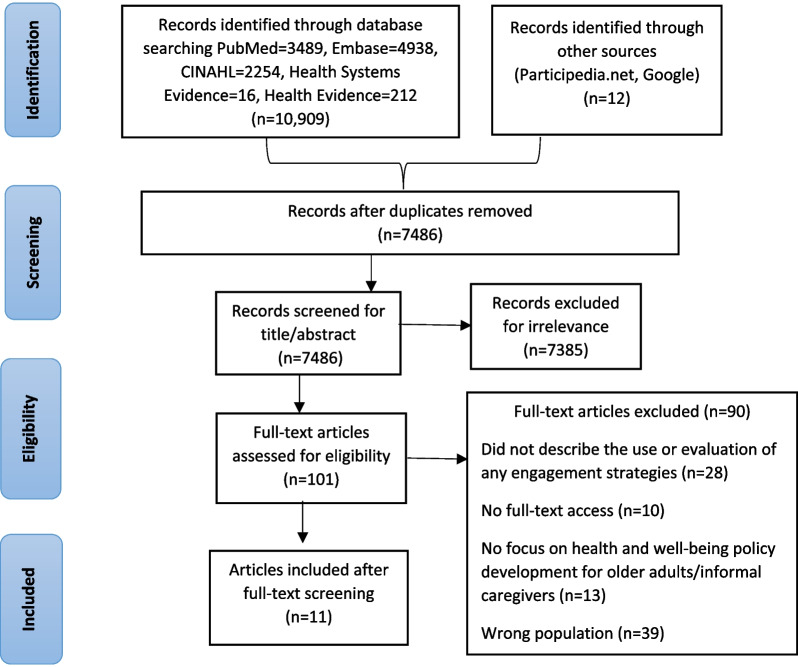
Preferred Reporting Items for Systematic Reviews and Meta-Analyses (PRISMA) diagram showing article selection process

The published and grey literature search yielded 10 921 publications. After removing duplicates, 7486 articles were included for the title and abstract screening. We excluded 7385 articles for irrelevance. Altogether, 101 articles were assessed for full-text eligibility, 90 articles were excluded and data were extracted from 11 publications (Fig. 1). We identified the engagement methods in the relevant articles and described how they were used to engage participants in health policy development. Then, we interpreted these findings on the basis of the elements in the framework.

### Study characteristics

The studies included were conducted in countries in North America [40–42] (*n* = 3), Europe [17, 43–45] (*n* = 4), Australia [46–48] (*n* = 3) and Asia [29] (*n* = 1). Publication dates of included studies ranged from 1997 to 2021. The design of the studies included a multi-method design [45] (*n* = 1), qualitative designs [17, 29, 40–43, 46, 47] (*n* = 8), a quantitative design [48]) (*n* = 1) and a case study design [44] (*n* = 1; Table 1). About half of the studies [17, 41, 44, 45, 47, 48] (*n* = 6) involved both older adults and their informal caregivers. Four studies [40, 42, 43, 46] involved only older adults, and one study [29] involved only informal caregivers in health policy development. A wide range of older adults (people with chronic illnesses, dementia, complex health and social needs, and home-bound older adults) and their informal caregivers were included in the studies. Finally, some engagement strategies were implemented as a one-time event, while others were ongoing or continued for a longer period of time.

**Table 1:** Characteristics of included studies

Author(s); country	Study objectives	Study type/design	Participants characteristics	Duration	Engagement strategies
Spiers et al., 2021; UK	To understand what matters to people living with multiple long-term conditions and identify priorities for future research	Multi-methods design	Older adults with multiple long-term conditions (age 80 years and older) and their informal caregivers (*n* = 130)	One-off (not clear)	Survey interview workshop
O’Keefe and Hogg, 1999; UK	To develop ways of reaching house-bound people and enabling them to give their views in planning and monitoring health and social care	Case study design	House-bound older adults (age 70 years and older) and their informal caregivers (*n* varied at different points in time throughout the project)	On-going (6 years). Focused on building infrastructure for user involvement	Survey Interview Group discussions Telephone conferencing
Keogh et al., 2021; Ireland	“To create a pathway for the voice and experiences of people with dementia and family caregivers to influence upcoming legislation on home caregivers currently represented through advocacy but not through direct voice using two innovative methods” [17]	Qualitative research design	Older adults with dementia (age 65 years and older; *n* = 10; 5 males, 5 females) and informal caregivers (age not reported; *n* = 28; 5 males, 23 females)^a^	One-off involvement but with previous years of discussion with people with dementia Policy cafe (2.5 h) Careers’ assembly (1 day)	Policy café carers’ assembly
Kaambwa et al., 2015; Australia	To provide empirical evidence on the features of consumer-directed care (CDC) most important to older adults and their informal caregivers	Quantitative research design	Older adults (mean age 80 years; *n* = 87; 75% females) and caregivers (mean age 74 years; *n* = 30; 53% females)	One-off (not clear)	Discrete choice experiment
Jowsey et al., 2011; Australia	To compare the recommendations for chronic illness care made in the National Health and Hospital Reform Commission (NHHRC) final report with suggestions made by people with chronic illness and family caregivers of people with chronic illness in a recent Australian study	Qualitative research design	Older adults with chronic illnesses (age 65 years and older; *n* = 52) and family caregivers (*n* = 14)^b^	One-off (40–90 min)	Semi-structured interviews
Harrison et al., 2021; UK	To explore the use of photo-elicitation to involve older adults on the issue of age-friendliness in a rural area	Qualitative visual methodologies	Older adults (age 60 years and older; *n* = 13; 10 females, 3 males)^c^	Ongoing (6 months). Authors reported that participants continued as co-researchers	Photo-elicitation
Gauvin et al., 2019; Canada	To engage older adults with complex health and social needs, and their caregivers, to improve hospital-to-home transitions	Qualitative facilitated panel discussions	Older adults with complex needs (*n* = 8) and their caregivers, (*n* = 4)^d^	One-off (1 day)	Citizen panel
Degeling et al., 2018; Australia	“To elicit informed views from Australian women aged 70–74 regarding the acceptability of ceasing to invite women their age to participate in government-funded mammography screening” [46]	Qualitative research design	Women aged 70–74 years with no personal history of breast cancer (*n* = 34)	One-off (2 days)	Community juries
Chuengsatianup et al., 2019; Thailand	“(1) To examine how public participation in health policy can be actualized through a citizens jury as an operational model (2) To understand the strengths and weaknesses of the ways in which the idea was made operational, and (3) To provide recommendations for further use of the model” [29]	Qualitative research design	Caregivers of older adults (age 35–75 years; *n* = 12; 5 females, 7 males)	One-off (4 days)	Citizens’ jury interviews Focus group discussions
Chappell, 1997; Canada	To understand views of older adults on Pharmacare reference-based policy	Qualitative research design	Focus group: older adults (age 65 years and older; *n* = 51; 30 females and 21 males Interviews: Older adults (age 65 years and older; *n* = 1699)	One-off (focus group not clear; telephone interviews 30 min)	Focus group discussions Telephone interviews
Buman et al., 2012; USA	To describe a method for engaging older adults in policy regarding food and physical activity	Qualitative visual methodology	Older adults (age 65 years and older; *n* = 9–12)	Ongoing (1 year and 3 months)	Photovoice and audio recordings

^a^7 out of 10 older adults were aged 65 years and older

^b^64% of the study participants were between 65 and 85 years and the remaining 36% were below 65 years

^c^Most of the participants were reported to be 65 years and older

^d^9 out of the 12 participants (9 females, 3 males) were 65 years and older

### Engagement strategies

A total of 13 unique engagement strategies were reported across the 11 included articles. We adopted the World Bank Groups’ categorization of citizen engagement being traditional consultation and feedback mechanisms, participatory mechanisms and citizen-led mechanisms [49]. For this review, we slightly adjusted these categories to traditional strategies, deliberative strategies, and other strategies (see Additional file 4: Appendix S4 for definition of terms). Traditional engagement strategies (interviews, surveys, focus groups, workshops, telephone conferencing) were categorized as such, as they are commonly used strategies not unique for engagement (for example, also used for research purposes) and generally designed to measure the prevalence and range of opinions and not their stability or depth [50, 51]. Deliberative strategies (citizen juries, citizen panels, community juries, policy café and carers assembly) were thus categorized, as they highlight participants’ prior education on the topic of discussion (for example, using citizen briefs and expert witnesses), thus eliciting informed views and perspectives on complex topics [50]. The “other strategies” category under which we classified a discrete choice experiment and two visual engagement strategies (photo elicitation and photovoice and audio recording) were so categorized under a general heading “other engagement strategies”, as they did not fit into the traditional or deliberative categories.

Traditional engagement strategies only (*n* = 5) were reported in four articles [42, 44, 45, 47], deliberative strategies only (*n* = 4) were reported in three articles [17, 41, 46], one article reported using one deliberative method and two traditional methods [29], and the remaining three articles [40, 43, 48] reported “other” engagement strategies. The strategies engaged participants in a wide range of health policy issues, most of which related to older adults. One article used carers assembly to engage caregivers of people with dementia to discuss issues of concern to them and to identify priorities to bring to policy-makers about to make important legislative decisions on the future provision of home care in Ireland [17]. Traditional engagement strategies were used to engage participants on recommendations for improving care for people with chronic illnesses (semi-structured interviews), priorities for older adults with multiple long-term conditions (survey, interview, workshop), quality of life and care in nursing homes, medication reimbursement (focus group, telephone interview), eldercare and informal caregiver policy (policy café and carers assembly) and health-related concerns of homebound people (survey, interview, group discussion, telephone conferencing). Deliberative methods were used to engage participants on government-funded mammography screening (community jury), long-term care provision (citizen jury with interview and focus group), post-diagnosis support/home care legislation (policy café and carers assembly) and hospital-to-home care transitions (citizen panels). Finally, other methods engaged participants in consumer-directed care (discrete choice experiment), age-friendliness of a rural community (photo-elicitation) and improving neighbourhood food and physical environment (photovoice and audio recording). Table 2 presents a summary of the engagement strategies, their descriptions and examples of health policy issues addressed.

**Table 2:** Categorization and description of methods for engaging older adults and informal caregivers in health policy development

Category name	Engagement method	Description	Examples of health policy issues*
Traditional engagement strategies	Survey interview workshop [45]	Surveys provide a quantitative assessment of older adults/informal caregivers’ views on health policy [49]. An interview is a conversation between an interviewer (who asks questions) and an interviewee (older adults and informal caregivers) who responds to the questions. For example: survey participants were enlisted through diverse channels such as social media, charities and networks, while interviewees were recruited from community services and care homes. This strategy aimed to ensure the inclusion of older individuals who might lack access to digital platforms, thereby broadening the reach to those who could be easily disregarded.	Priorities for older adults with multiple long-term conditions [45]^*^ Health inequalities and potential policy response [68]
	Semi-structured interviews [47]	Semi-structured interviews are a conversation between an interviewer and an interviewee (older adults and informal caregivers) based on a semi-structured interview guide, which is a schematic presentation of questions or topics to be explored by an interviewer. For example, 40–90 min interviews followed by a 10-min survey on personal details were conducted to understand how older adults experience the management of chronic illnesses and how the Australian healthcare system could be improved.	Improving care for people with chronic illnesses [47]
	Focus groups Telephone interviews [42]	Focus groups are composed of a small number (usually 3–8) of older adults and/or informal caregivers to gain a detailed understanding of their perspectives, values and concerns [49]. Participants are interviewed in a discussion setting facilitated by a moderator [69]. For example, seven focus groups of older adults were organized to discuss views on the content and implementation strategies of Pharmacare policies. These were followed by brief telephone interviews with 1699 older adults to ascertain the generalizability of the findings emerging from the focus group.	Medication reimbursement/Pharmacare’s Reference-Based Pricing (RBP) policy [42]^*^ Consumer perception of food-related risks [70]
	Survey interview group discussion Telephone conferencing [44]	Surveys (via postal questionnaires), telephone conferencing (four or five members with a facilitator discuss a particular topic of interest), group discussions and face-to-face interviews in participants’ home were used to reach older adults and informal caregivers who would otherwise be precluded. For example, homebound people and their informal caregivers were involved in planning and monitoring health and social care using community development techniques. They were identified through leaflets, targeted media coverage, and agencies in touch with them.	Health-related concerns of homebound people/quality of life/planning and monitoring health and social care [44]^*^
Deliberative engagement strategies	Community juries [46]	A community jury is a group of citizens (older adults and informal caregivers) brought together to receive detailed evidence about and deliberate on a specific (health policy) issue [53]. The term “community jury” is sometimes used interchangeably with “citizen jury”. [71] For example, two community juries were held over 2 days. Day 1: Prerecorded testimonies from four experts were presented. Day 2: Discussion and debating of evidence and presentation of recommendations.	Government-funded mammography screening [46]^*^
	Citizens’ jury interviews Focus groups [29]	Citizens’ juries are a group of selected members (for example, informal caregivers of older adults, usually 12–24) of a community that make recommendations to supplement conventional democratic processes [49]. This deliberative method was used alongside two traditional methods: interviews and focus groups which were conducted randomly with jurors and who at the end of the workshop evaluated and reflected on their experiences [29]. For example, during a 4-day workshop in Bangkok, 12 jurors from five regions participated. The first day involved orientation and the sharing of experiences by family caregivers. On the second and third days, expert witnesses provided briefings on community-based and institution-based long-term care, respectively. The final day included a voting session on resource allocation among 10 policy programs for long-term care. Additionally, informal semi-structured interviews (15–45 min) were randomly conducted with jurors, and focus groups were held at the end of the workshop for jurors to evaluate and reflect on their experiences.	Eldercare policy/long-term care policy [29]^*^ Mammography screening [72]^*^
	Citizen panel [41]	A citizen panel is a deliberative dialogue approach that provides the opportunity for older adults and their informal caregivers to make informed judgments about addressing high-priority issues on the basis of their values and preferences [73]. For example, a citizen panel brought together older adults with complex care needs and their caregivers to elicit their views on improving hospital-to-home transitions after providing them with a citizen brief (evidence from research that served as a basis for the discussions in the citizen panel). They discussed the underlying problem, possible elements to address it and potential barriers and facilitators to implement these elements.	Transitions of care [41]^*^ Health technology assessment processes [74]
	Policy café carers assembly [17]	Policy café and carers’ assembly are citizen engagement and organizational change process tools which emphasize a comfortable environment in a less formal setting. They emphasize generating an informal and hospitable space where older adults and caregivers’ values and insights are important. Policy café was adapted from the world café – a participatory method for citizen engagement [75] – and carers assembly was adapted from citizens assembly [76]. For example, a policy café was held in a hotel venue, two tables with five participants each, a facilitator for each table, and 2.5 h duration; two tables discussed the two different topics and afterwards participants swapped the tables. Also, a carers´ assembly was convened as a day-long meeting in a hotel room, participants were arranged in round tables of 5–6 persons with a facilitator at each table, four brief presentations on the topic were followed by questions session and each topic was then considered separately in a round table discussion, voting on main issues based on discussion.	Home care legislation and post-diagnosis support [17]^*^ Challenges and opportunities of an ageing population [76]
Other engagement methods	Discrete choice experiment (DCE) [48]	A DCE is a survey instrument that clearly explains both a baseline or status quo situation and alternatives that can be chosen [77]. For example, a DCE was used to assess older adults’ preference of a consumer directed care (CDC) approach to consumer assisted care service (CACS) delivery. The questionnaires were researcher administered within a structured DCE and completed individually in a common venue. Each of the nine group exercises consisted of at most 20 older adults with the same 3–4 researchers available to facilitate the session and address any questions.	Consumer-directed care approach [48]^*^ Primary healthcare services among older adults with chronic disease [78]
	Photo-elicitation [43]	Photo elicitation is the insertion of a photograph by the researcher into a research interview to evoke information, feelings and memories owing to the photograph’s particular form of representation [79]. For example, photo-elicitation was used to explore the age-friendliness of a rural area in Northern England. Participants attended two sessions. The initial session was for information and training in photo-elicitation. In the second session, the photographs from each of the group participants were viewed and discussed.	Age-friendliness of a rural community [43]^*^ Inclusion in education [79]
	Photovoice and audio recordings [40]	Photovoice is a process by which citizens identify, represent and enhance their community through a photographic technique. Its three main goals are to enable people to record and reflect on their community’s strengths and concerns, to promote dialogue about community issues through group discussions of photographs and to reach policy-makers. For example, older adults used photovoice to document features of the food and physical environment and then used audio recording devices to develop audio narratives about their observations. These were discussed in community action teams (CATs) meetings made up of 9–12 older adults.	Improving neighbourhood food and physical environment [40]^*^ Respect and social inclusion in cities [80]

^*^These examples are from studies with target population of older adults aged 65 years and above and informal caregivers. Other examples are from studies applying to the general population or different age groups. Examples under description in column 3 are from the included studies

### *Synthesis of result according to Carman *et al.*’s framework*

#### Continuum of engagement

**Fig. 2:**
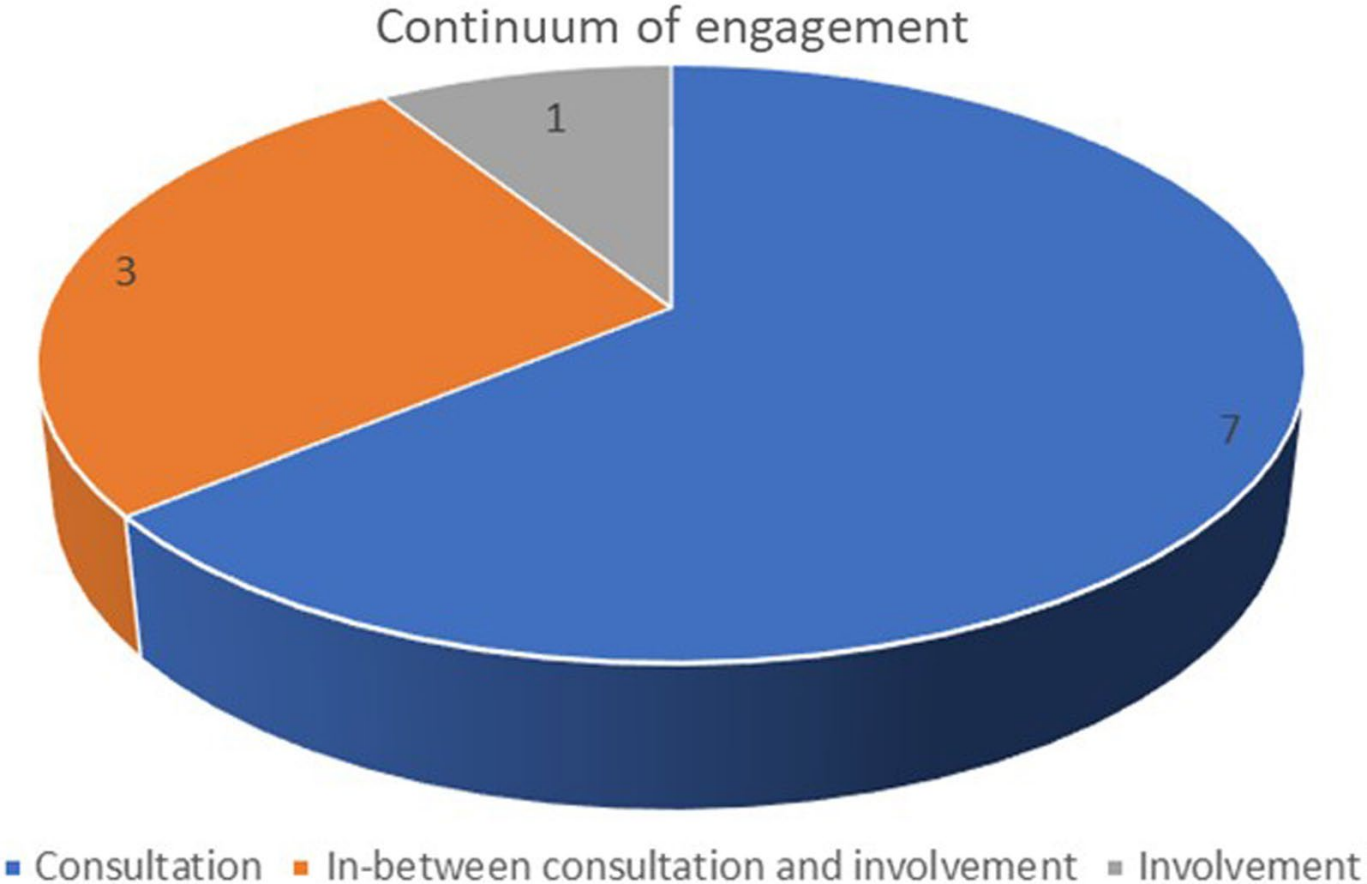
Distribution of studies (*n* = 11) over the continuum of engagement

Identified engagement strategies varied in the level of influence (engagement continuum) participants had, with no clear relationship between engagement approach and continuum of involvement. In all, 4 of the 11 included articles reported engagement approaches on the lower end of the engagement continuum (that is, views on and experiences with a health issue were elicited [45–48]. Six articles [17, 29, 40–43] reported engagement approaches best situated in between the consultation and involvement continuum (that is, in addition to simply eliciting views and experiences, participants’ opinions were used for advocacy, development and presentation of research evidence and policy recommendations to policy-makers). This is a newly added component to the Framework. Finally, one article [44] reported engagement strategies with characteristics fitting the involvement continuum (that is, older adults’ inputs to contributed to changes in service provisions). No article reported engagement strategies reflecting participants’ involvement on the highest end of the continuum (partnership/shared leadership; Fig. 2). The level of power and decision-making authority that older adults and informal caregivers had was not a function of the engagement approach/category. Thus, it was impossible to establish a clear relationship between individual engagement approaches/the category they belong to, and the engagement continuum. For example, three unique traditional engagement strategies (interviews, surveys, and workshops) belonged to the consultation continuum, thus indicating that older adults/caregivers involved using those approaches had a lower level of power in decision-making. Contrastingly, another article described similar traditional engagement strategies (survey, interviews, group discussions and telephone conferencing), but older adult and informal caregivers involved had a higher level of influence in decision-making (involvement continuum) as shown in Table 3.

**Table 3:** Strategy category by continuum of engagement

Strategy category	Continuum of engagement		
	Consultation	In-between consultation and involvement	Involvement
Traditional strategies	Survey Interview Workshop [45]	Focus group Telephone interviews [42]	Survey Interview Group discussion Telephone conferencing [44]
	Semi-structured interviews [47]		
Deliberative strategies	Community jury [46]	Policy café and carers assembly [17]	0
		Citizen juries Interviews Focus groups [29]	
		Citizen panels [41]	
Other strategies	Discrete choice experiment [48]	Photovoice and audio-recording [40]	0
		Photo elicitation [43]	

### Stages of policy development

Regarding the stages of policy development, none of the 11 studies described older adults and their informal caregivers’ involvement beyond the agenda/priority-setting stage of policy development. Their role in the process of policy formulation, implementation and evaluation was not clear. Most of the studies reported participant involvement in identifying health priority issues or care needs and examining how citizen involvement can be actualized. Also, no studies reported on factors influencing policy-makers to create engagement opportunities.

### Outcomes of engagement

Of the 11 studies, 8 reported on outcomes of older adult/informal caregiver engagement in policy development. Outcomes reported in the studies included but were not limited to developmental outcomes (civic education of citizens, citizens’ developed capacity to participate in public policy issues) [29, 45, 46] and instrumental outcomes (promotion of active citizenship and awareness of lived experiences of other older adults) [17, 29]. Other outcomes included increased health and information access, quality of life and self-esteem of participants [44]; provision of reform solutions [47]; and priorities for policy-makers [17]. Finally, some engagement strategies were reportedly used in isolation [41, 43, 46–48], while others were combined with one [17, 40, 42, 45] or more than one [29, 44] engagement method.

## Discussion

This review synthesized existing literature on strategies for engaging older adults and informal caregivers in health and well-being policy development. Findings suggest that, although older adults and informal caregivers were consulted for identifying priorities and to elicit their opinions on health issues, they were rarely engaged in the actual processes of policy formulation, implementation and evaluation. They also rarely had shared leadership and decision-making authority. None of the included studies provided data on factors influencing policy-makers to create engagement opportunities and data on comparisons of alternative engagement strategies for variation, content, breadth and depth of participants’ input.

Our categorization of engagement methods into “traditional”, “deliberative” and “others” is in line with previous research suggesting the same. For example, the World Bank categorized engagement mechanisms as traditional and consultative feedback mechanisms (including surveys and focus groups), participatory mechanisms and citizen-led mechanisms. Similarly, approaches such as citizen juries, community juries, citizen panels and policy cafés have been described as deliberative engagement methods in previous literature, usually characterized by the presenting research evidence or using expert witnesses to educate lay citizens to make reasoned and informed judgement on complex issues [17, 50, 52, 53]. Finally, photovoice and audio-recording and photo elicitation, categorized under the other category with the discrete choice experiment, have been described in previous research as visual research methodologies [54, 55].

Furthermore, findings from this review indicate that the engagement of older adults in policy-making often relies solely on consultation approaches [15] and takes place at the beginning stages of the policy cycle through older adult and informal caregiver consultation and priority identification. However, meaningful participation involves stakeholders in all stages of the policy cycle. This includes research, data collection, priority setting, policy formulation, budgeting, implementation and review and evaluation [15, 56]. Thus, future research can be conducted to increase our understanding of older adult and informal caregiver involvement in the higher end of engagement continuum as well as how or when in the policy cycle/process they are involved.

Most of the engagement strategies reported in this review involved participants directly and not through advisory bodies or organizations of older persons, although some collaborations and partnerships with other stakeholders were necessary in some cases to reach the older adults and their informal caregivers [17, 40, 43, 44]. Also worthy of note is the fact that leveraging on partnerships, institutionalizing engagement and community-based partnerships is critical to enabling desired engagement outcomes [12, 40, 43, 44]. Finally, the literature on engagement strategies for other populations can be useful to inform future empirical work on engagement strategies for older adults aged 65 years and above. These strategies include the participatory theatre approach [57], concept maps [58], deliberative polling and citizen dialogues [59].

Although our research focuses solely on identifying literature that describes the use and evaluation of older adults and informal caregivers involvement approaches, we recognize that older persons or informal caregivers’ participation in policy-making can take place both in individual and collective settings [60]. Thus, there may be other engagement approaches that accommodate older adults and informal caregivers in group settings with other stakeholders and not as the sole participants, for example, public consultations on the living conditions of seniors which involved older adults, representatives of health and social services, and elected members of city councils [61]. The choice of engagement approach may be dependent on existing policy processes within a particular setting, for example, senior councils in Europe for supporting local political decision-making. Also, Keogh and colleagues [17] designed innovative methods for involving people with dementia in policy development, using a world café methodology and citizen assembly model commonly implemented in Ireland [17, 62].

The evaluation of engagement methods has been minimal. Understanding the effectiveness of engagement strategies is fundamental to informing the design of successful engagement and reaching the full potential of engagement [16, 63–65]. This review discusses existing engagement strategies, but it does not address which may be most effective for older adults and informal caregivers based on the context/health system. One of the excluded studies (on the basis of the age criterion) reported on the evaluation of engagement strategies. Two deliberative methods of public participation – the citizens’ workshop and the citizens’ jury – were evaluated. The evaluation of the methods was based on process and outcome evaluation measures [66], and found that both methods were not rated significantly differently by the participants on most criteria, thus, signalling an overlap in the impact and utility of the two methods of deliberation. There is need for more studies on the evaluation of engagement strategies for involving older adults 65 years and older and their informal caregivers in health policy development.

Having established that research is needed around the evaluation of engagement methods, we also need to provide the older adults and informal caregivers with the necessary skills needed to educate themselves about the process of policy-making as well as advocacy training. Engaging them in advocacy has the potential to achieve better local policy outcomes [40]. For example, one excluded study (on the basis of the age criterion) reported the evaluation of a senior civic academy (SCA) methodology: a self-advocacy course that simultaneously educates older residents about policy-making processes and engages them in advocacy training to incorporate their voices in local policy and planning. A pre- and post-program evaluation, as well as follow-up interviews, were conducted [67]. The study reported the efficaciousness of the methodology in engaging older adults. Advocacy training can be provided for older adults and informal caregivers on advocating for policy changes. Training components may include building a case for change, identifying potential allies and resources, and role-playing scenarios targeting local policy-makers [40]. Finally, civil society organizations and older adult organizations also play a role in building the skills of older persons to engage in advocacy with governments, thus creating a space for them to tell their own stories from their perspectives to inform policy-making [56].

### Strengths and limitations

This is the first attempt we can identify to synthesize evidence on strategies for engaging older adults and informal caregivers in health policy development. We used the Multidimensional Framework for Patient and Family Engagement in Health and Healthcare to guide data extraction and analysis, thus providing a theoretical contribution to the literature.

Some limitations were, however, identified. First, although the review was not sufficient to provide information to build on the framework (for example, it was not clear how or whether older adults and their informal caregivers are involved in all the stages/cycle of policy development; also no factors influencing policy-makers to create opportunities for involvement were identified), gaps in the literature were identified, providing direction for future studies. Secondly, due to the ambiguity and heterogeneity of terminology for engagement, the search string for this review, although built with the help of librarians, may not have identified all relevant published and grey literature. Quality appraisal, which is an evaluation of the quality of the included publications, was not performed, since our objective was to identify and describe the range of literature available on the topic without excluding studies on the basis of methodological quality. Furthermore, our intention was to provide a comprehensive overview of the field rather than to evaluate the quality of individual studies. Also, quality appraisal is not required in scoping review methods. Finally, our research focused solely on engagement approaches that were used exclusively for older adults and informal caregivers; thus, we may have missed out on publications reporting approaches that include other citizens and stakeholders alongside older adults and informal caregivers.

### Implications for future research and policy

An examination of the existing literature points to a dearth of literature on the use and evaluation of engagement strategies for older adults aged 65 years and above and their informal caregivers. Implications for future research include understanding different healthcare systems and contexts in which these methods were used, understanding factors influencing policy-makers to create engagement opportunities for them and researching methods for evaluating and assessing engagement strategies for variation, content, breadth and depth of participants’ input.

Policy-makers can learn from the findings of this review by understanding how older adults and informal caregivers can be involved in policy process using the identified approaches. Engaging older adults and their informal caregivers in health policy formulation, implementation and evaluation beyond just consultations and tokenism [33] but also, in the continuum of involvement and partnership/shared leadership, requires a concerted effort of all stakeholders, including researchers, policy-makers, older adults’ representative organizations and older adults and their informal caregivers.

## Conclusion

The analysis shows a dearth in literature on the use and evaluation of strategies for engaging older adults and informal caregivers in health policy development. The small number of studies reviewed could indicate a need to further explore older adult and informal caregiver involvement in collective settings including other stakeholders. Finally, this review highlights gaps in strategies for involving older adults and informal caregiver, and provides relevant information to enable policy-makers to make evidence-informed and responsive policy decisions, thus improving health outcomes.

### Supplementary Information

**Additional file 1: Appendix S1.** A Multidimensional Framework for Patient and Family Engagement in Health and Healthcare by Carman et al. [25].

**Additional file 2: Appendix S2.** Search strategy.

**Additional file 3: Appendix S3.** Data charting table.

**Additional file 4: Appendix S4.** Definition of terms.

## Data Availability

All data are publicly available and included within the article and its supplementary information files.
