# Supplementary material for: Strategies for engaging older adults and informal caregivers in health policy development: A scoping review
Source: Health Res Policy Syst. 2024 Feb 19;22:26. doi: 10.1186/s12961-024-01107-9 (PMC10875823; doi:10.1186/s12961-024-01107-9)

**Strategies for engaging older adults and their informal caregivers in health policy development.**

**Additional file 1**

**Appendix S1: A Multidimensional Framework for Patient and Family Engagement in Health and Healthcare by Carman et al [25].**


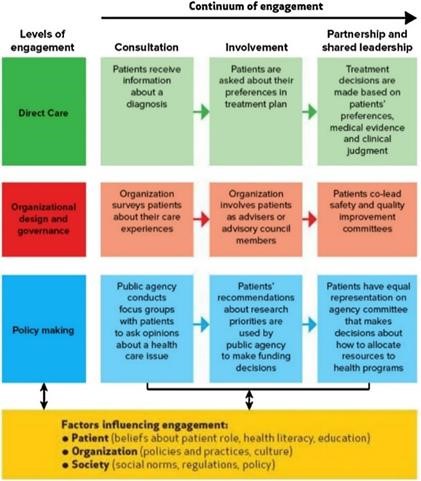

Supplement: Supplementary file 1 — Additional file 1: Appendix S1. A Multidimensional Framework for Patient and Family Engagement in Health and Healthcare by Carman et al. [25]. [file 12961_2024_1107_MOESM1_ESM.docx]
