# Supplementary material for: Strategies for engaging older adults and informal caregivers in health policy development: A scoping review
Source: Health Res Policy Syst. 2024 Feb 19;22:26. doi: 10.1186/s12961-024-01107-9 (PMC10875823; doi:10.1186/s12961-024-01107-9)
Supplement: Supplementary file 2 — Additional file 2: Appendix S2. Search strategy. [file 12961_2024_1107_MOESM2_ESM.docx]

**Strategies for engaging older adults and their informal caregivers in health policy development.**

**Additional file 2: Appendix S2: Search strategy**

| **Database** | **Concept** | **Search terms** |
| --- | --- | --- |
| **PUBMED** | Older adult | ("Aged"[Mesh] OR “aged patient*”[tiab] OR “aged people”[tiab] OR “aged person*”[tiab] OR “aged adult*”[tiab] OR “aged citizen*”[tiab] OR elder*[tiab] OR “oldest old”[tiab] OR “older adult*”[tiab] OR “older patient*”[tiab] OR “older subject*”[tiab] OR “older citizen*”[tiab] OR “older person*”[tiab] OR “older people”[tiab] OR senior*[tiab] OR “old age”[tiab] OR “advanced age”[tiab] OR aging[tiab] OR ageing[tiab] OR "Geriatrics"[Mesh] OR geriatr*[tiab] OR gerontolog*[tiab] OR Centenarian*[tiab] OR Nonagenarian*[tiab]  OR Octogenarian*[tiab] OR septuagenarian*[tiab]) |
|  | Engagement | "Patient Participation"[Mesh] OR participat*[tiab] OR Empower*[tiab] OR Activat*[tiab] OR Deliberat*[tiab] OR engag*[tiab] OR involv*[tiab] OR "Stakeholder Participation"[Mesh]  OR “stakeholder role*”[tiab] |
|  | Health policy development | "Health Policy"[Mesh] OR “health polic*”[tiab] OR “healthcare polic*”[tiab] OR “health care polic*”[tiab] OR "Policy  Making"[Mesh] OR “policy making”[tiab] OR “policymaking”[tiab] OR “policy development*”[tiab] OR “policy analys*”[tiab] OR “advisory committ*”[tiab] OR “task force*”[tiab] OR “review commit*”[tiab] OR “policy formulation*”[tiab] OR “policy evaluation*”[tiab] OR “health care reform*”[tiab] OR “healthcare reform*”[tiab] |
