# Supplementary material for: Strategies for engaging older adults and informal caregivers in health policy development: A scoping review
Source: Health Res Policy Syst. 2024 Feb 19;22:26. doi: 10.1186/s12961-024-01107-9 (PMC10875823; doi:10.1186/s12961-024-01107-9)
Supplement: Supplementary file 3 — Additional file 3: Appendix S3. Data charting table. [file 12961_2024_1107_MOESM3_ESM.docx]

**Strategies for engaging older adults and their informal caregivers in health policy development.**

**Additional file 3: Appendix S3: Data charting table**

| **Scoping Review Details** | |
| --- | --- |
| Article title |  |
| Review objectives |  |
| Review questions |  |
| Study type (case, empirical, review) |  |
| Study design (quantitative, qualitative) |  |
| **Evidence source details and characteristics** |  |
| Citation details |  |
| Country/geographical setting |  |
| Context |  |
| Participants/population studied (age, sex, number) |  |
| **Inclusion/Exclusion Criteria** | |
| **Population**  Includes older adults aged 65 and above and/or their informal caregivers |  |
| **Concept**  Describes strategies for older adult and informal caregiver engagement |  |
| **Context**  Describes strategies for older adult and informal caregiver engagement in policies around health and well-being |  |
| **Details/results extracted from publications** | |
| Name of engagement strategies discussed |  |
| Description of strategy |  |
| Stated continuum of engagement (consultation, involvement, partnership, and shared leadership) |  |
| Stated phase of policy development (e.g. policy formulation, implementation, or evaluation) |  |
| Stated factors influencing reported engagement strategy |  |
| Stated outcome(s) of engagement strategies E.g.,  Change in knowledge and attitude of engaged older adults and informal caregivers  Promotion of active citizenship (e.g., do older adults and informal caregivers feel a sense of citizenship and participation in decision-making)  Impact on the relationship between the government and the citizens  Older adults’ and informal caregivers' awareness of one another’s lived experiences.  Increased knowledge about care transitions and engagement  Developed capacity to take part in public policy matter  Any other reported outcomes |  |
| Data on comparisons of different engagement approaches |  |
| Data on efforts/initiatives to ensure engagement of older adults of minority groups, if stated |  |
| Topics discussed using engagement approaches |  |
|  |  |
