# Supplementary material for: Strategies for engaging older adults and informal caregivers in health policy development: A scoping review
Source: Health Res Policy Syst. 2024 Feb 19;22:26. doi: 10.1186/s12961-024-01107-9 (PMC10875823; doi:10.1186/s12961-024-01107-9)
Supplement: Supplementary file 4 — Additional file 4: Appendix S4. Definition of terms. [file 12961_2024_1107_MOESM4_ESM.docx]

**Strategies for engaging older adults and their informal caregivers in health policy development.**

**Additional file 4: Appendix S4: Definition of terms**

| **Term** | **Definition** |
| --- | --- |
| Citizen | Every person in a society or country in an all-encompassing way without discrimination. |
| Older adults | People aged 65 years and older. |
| Informal caregiver | An individual who provides care, not as a primary occupation (for example, a spouse, partner, family member, friend, or neighbor) and is involved in assisting with activities of daily living and medical tasks. |
| Citizen engagement | Citizen engagement is the meaningful involvement of individual citizens in policy or program development, from agenda-setting and planning to decision-making, implementation, and review. It requires two-way communication that is interactive and iterative with an aim to share decision-making power and responsibility for those decisions. |
| Consultation | Eliciting opinions from older adults and informal caregivers using methods such as focus groups, surveys, etc. |
| Involvement | Using recommendations made by older adults and their informal caregivers to inform public decisions. |
| Partnership/shared leadership | Equal opportunities for older adults and their informal caregivers to be on decision-making committees and contribute to policy development. |
| Traditional engagement strategies | Commonly used methods not unique for engagement (e.g., also used for research purposes), and generally designed to measure the prevalence and range of opinions and not their stability or depth. |
| Deliberative methods | Engagement methods that highlight ’participants' prior education on the topic of discussion (for e.g., citizen briefs and expert witnesses) and engagement in new knowledge, thus eliciting informed views and perspectives on complex topics. |
| Other engagement methods | Other engagement methods are those that do not fit into the categories of traditional and deliberative. For these articles they were 2 visual engagement methods, and a Discrete Choice Experiment. |
| Outcomes of engagement | Effect or result of engaging older adults and their informal caregivers in health policy development. |
| Evaluation of engagement strategies | Effectiveness of methods for engaging older adults and informal caregivers in health policy development to produce the impact of engagement on policy decision-making. |
